# Evolution of soccer talent identification criteria: a systematic review from global perspectives (1976–2024)

**DOI:** 10.3389/fpsyg.2026.1739010

**Published:** 2026-03-19

**Authors:** Yikang Gong, Weichao Jiang, Yuan Li, Jinrong He, Wei Zhang, Wenzhe Li, Shiqin Chen, Chong Luo

**Affiliations:** 1 Shanghai University of Sport, Shanghai, China; 2 Beijing Sport University, Beijing, China

**Keywords:** cross-cultural adaptation, dimensional evaluation, dynamic evaluation, selection criteria, soccer talent identification

## Abstract

**Introduction:**

This systematic review synthesizes the evolution of indicators used in global soccer talent identification (TID) between 1976 and 2024, with particular emphasis on how technological innovation, policy frameworks, and cultural contexts have shaped selection systems. In the context of accelerating globalization, TID criteria have progressively shifted from predominantly subjective, experience-based judgments to multidimensional and integrative evaluation models.

**Methods:**

A systematic review of empirical research published from 1976 to 2024 was conducted. Thirty-eight empirical studies meeting predefined inclusion criteria were synthesized to map historical shifts in TID indicators and to develop an evolutionary framework describing changes in identification practices over time.

**Results:**

The reviewed evidence supports a three-stage progression in soccer TID indicator development. The early stage (1976–1999) was dominated by anthropometric measures and basic physical fitness assessments. The developmental stage (2000–2015) featured the integration of technical performance metrics with physiological testing, reflecting expanding methodological capacity and sport-science uptake. The active stage (2016–2024) was characterized by multidimensional integration, with increased incorporation of psychological attributes and social adaptability alongside physical, technical, and physiological domains. Across stages, technological advancement and intensifying global competition emerged as major drivers of indicator expansion and methodological refinement. Tools such as wearable sensor systems, GPS tracking, and multidimensional assessment frameworks broadened the scope of quantifiable indicators and promoted more standardized evaluation workflows. Europe has been particularly influential in advancing standardization and disseminating related practices internationally. However, the review also demonstrates persistent regional differentiation: European systems generally emphasize data-driven precision and procedural standardization, whereas South American and African contexts more often prioritize the detection of latent potential and context-dependent performance under game-like conditions.

**Discussion:**

Global soccer TID indicators have evolved toward increasingly comprehensive, technology-enabled assessment systems, yet the trajectory of development remains shaped by regional philosophies of talent and contextual constraints. The findings underscore that effective TID systems should balance standardization with contextual sensitivity, integrating robust measurement with an appreciation of culturally embedded selection logics and ecologically valid performance demands.

## 1 Introduction

As one of the most influential sports worldwide, soccer not only reflects a nation’s athletic capacity but is also deeply intertwined with its commercial value and cultural identity (Butcher and Bryant, 2024). In contemporary competitive soccer, scientifically grounded talent identification and development (TID) programs form the foundation of elite performance systems, with their methodological rigor directly influencing youth training efficiency and the long-term competitiveness of professional leagues (Lüdin et al., 2023).

Since the 1970s, accelerating globalization has progressively transformed soccer talent selection from reliance on subjective experience toward multidimensional and evidence-informed evaluation. This transition has been shaped by the interaction of technological innovation, sociocultural traditions, and policy frameworks (Carlsson-Wall et al., 2024). While technological progress has promoted increasing standardization in assessment practices, it has also generated practical tensions arising from regional disparities in development models, institutional structures, and access to resources. As a result, talent identification criteria continue to vary substantially across countries and regions. Such heterogeneity not only challenges the fairness and effectiveness of selection processes but also contributes to the loss of potentially elite players who fail to progress within misaligned or restrictive identification systems. Accordingly, understanding global patterns in the evolution of soccer TID indicators has emerged as a pressing theoretical and methodological issue (Platvoet et al., 2023; Yin et al., 2024).

Existing research on soccer TID has largely concentrated on specific regions, age groups, or limited time periods, often without a systematic examination of long-term trends under conditions of globalization. Although international organizations such as the Fédération Internationale de Football Association (FIFA) have advocated for unified or harmonized selection standards (Robinson et al., 2024), fragmented identification systems persist due to enduring cultural differences, institutional traditions, and unequal resource distribution (Ju et al., 2025; Morganti et al., 2025). At the same time, rapid technological advancement has enhanced the precision and efficiency of talent assessment, while simultaneously introducing new risks. The growing reliance on biomechanical analyses and artificial intelligence (AI)–driven prediction models has reinforced an emphasis on quantifiable indicators, potentially marginalizing non-measurable yet performance-critical attributes such as creativity, perceptual–cognitive skill, and adaptive decision-making (Mannix et al., 2025).

Moreover, current conceptual frameworks have yet to adequately capture the multidimensional, context-dependent, and dynamic nature of soccer talent identification in a globalized environment. Cross-cultural exchange and evolving competitive demands continue to reshape selection logics, yet empirical evidence explaining how and why TID indicators change over extended periods remains fragmented. Against this backdrop, a systematic examination of the global trajectory of soccer TID indicators from 1976 to 2024 offers an opportunity not only to document historical patterns of change but also to elucidate the technological, policy-related, and sociocultural mechanisms that drive regional differentiation in selection practices.

By integrating cross-national longitudinal evidence and developing a dynamic analytical framework for soccer TID indicators, this study aims to address existing gaps in globalization-informed perspectives and long-term trend analysis. This integrative approach is expected to generate actionable implications for optimizing youth development systems, with particular relevance for developing soccer nations. Specifically, this study seeks to: (1) examine the complementarity and potential compatibility between European data-driven models and South American creativity-oriented approaches in support of context-sensitive innovation; (2) delineate the functional boundaries of technological tools and mitigate assessment biases associated with uncritical “technology-first” adoption; and (3) propose an inclusive and adaptive global TID framework that accommodates cultural diversity while supporting the sustainable competitiveness of soccer systems (Table 1).

**Table 1:** Topic search query.

Set search query	
#1	TS = (soccer OR football OR “association football”)
#2	TS = (“talent identif” OR “player select” OR “athlete assess” OR “scout system” OR “youth develop”)
#3	(#1) AND (#2)
#4	(#3) AND TS = (model* OR framework* OR criter* OR indicator* OR predict* OR evaluat*)

## 2 Methods

### 2.1 Search strategy

Studies retrieved global research evidence on soccer talent identification indicators through systematic literature searches conducted across major English-language databases, including Web of Science, PubMed, EBSCO, Scopus, and SportDiscus. In accordance with the PRISMA statement, Boolean logic was applied to combine key terms such as “soccer talent identification,” “selection criteria,” and “talent indicators” (Takkouche and Norman, 2011). The search strategy was restricted to peer-reviewed English-language publications available up to December 2024.

### 2.2 Eligibility criteria

The studies included in this review met the following inclusion criteria: (1) studies focused on soccer talent identification (TID) or selection indicators; (2) empirical research, policy documents, expert consensus reports, longitudinal tracking studies, or case analyses; (3) reliable data sources with comprehensive variable descriptions; and (4) full-text availability in English.

The exclusion criteria were: (1) studies related to non-soccer sports; (2) theoretical reviews, conference abstracts, short-term cross-sectional studies, or literature lacking raw data; and (3) duplicate publications or untraceable data sources.

### 2.3 Study quality and coding

Two researchers independently evaluated the methodological quality of the included studies using the quality criteria (Sarmento et al., 2018). Any discrepancies were resolved through discussion until consensus was achieved. Articles were assessed based on purpose (item 1), relevance of background literature (item 2), appropriateness of study design (item 3), sample studied (items 4 and 5), use of informed consent procedure (item 6), outcome measures (item 7 and 8), method description (item 9), significance of results (item 10), analysis (item 11), practical importance (item 12), description of dropouts (item 13), conclusions (item 14), practical implications (item 15), and limitations (item 16). All 16 quality criteria were scored on a binary scale (0/1).

### 2.4 Data extraction

Data extraction was independently performed by two reviewers using a standardized protocol to ensure accuracy and consistency. For each eligible study, key information was systematically collected, including the study title, authors, year of publication, sample size, study design, geographical location, and talent identification criteria. Detailed records were made of the specific indicators applied in soccer talent identification, such as physical fitness, technical skills, psychological attributes, and social adaptability, as well as any technological tools utilized (e.g., GPS, wearable sensors, or artificial intelligence–based systems). Information on data collection and analytical methodologies was also extracted, encompassing the types of tests, surveys, or devices employed and the analytical techniques applied. In addition, the principal findings, conclusions, and reported limitations of each study were documented, with particular attention to their implications for global TID models, regional variations, and the role of technological advancement. Any discrepancies between reviewers were resolved through discussion, and when necessary, corresponding authors were contacted to clarify unclear information. This rigorous extraction process was intended to ensure the inclusion of high-quality studies and reliable data for the review.

## 3 Results

### 3.1 Study selection

A total of 7,186 records were initially retrieved from the selected databases. The first step involved removing duplicate entries, resulting in the exclusion of 3,654 records, leaving 3,556 unique records for further screening. Subsequently, a title and abstract screening process was conducted, during which 3,472 records were excluded due to their irrelevance to soccer talent identification (TID) or failure to meet other predefined inclusion criteria. These records were either unrelated to the topic, non-empirical in nature, or did not fulfill the criteria established for this review.

**Figure 1:**
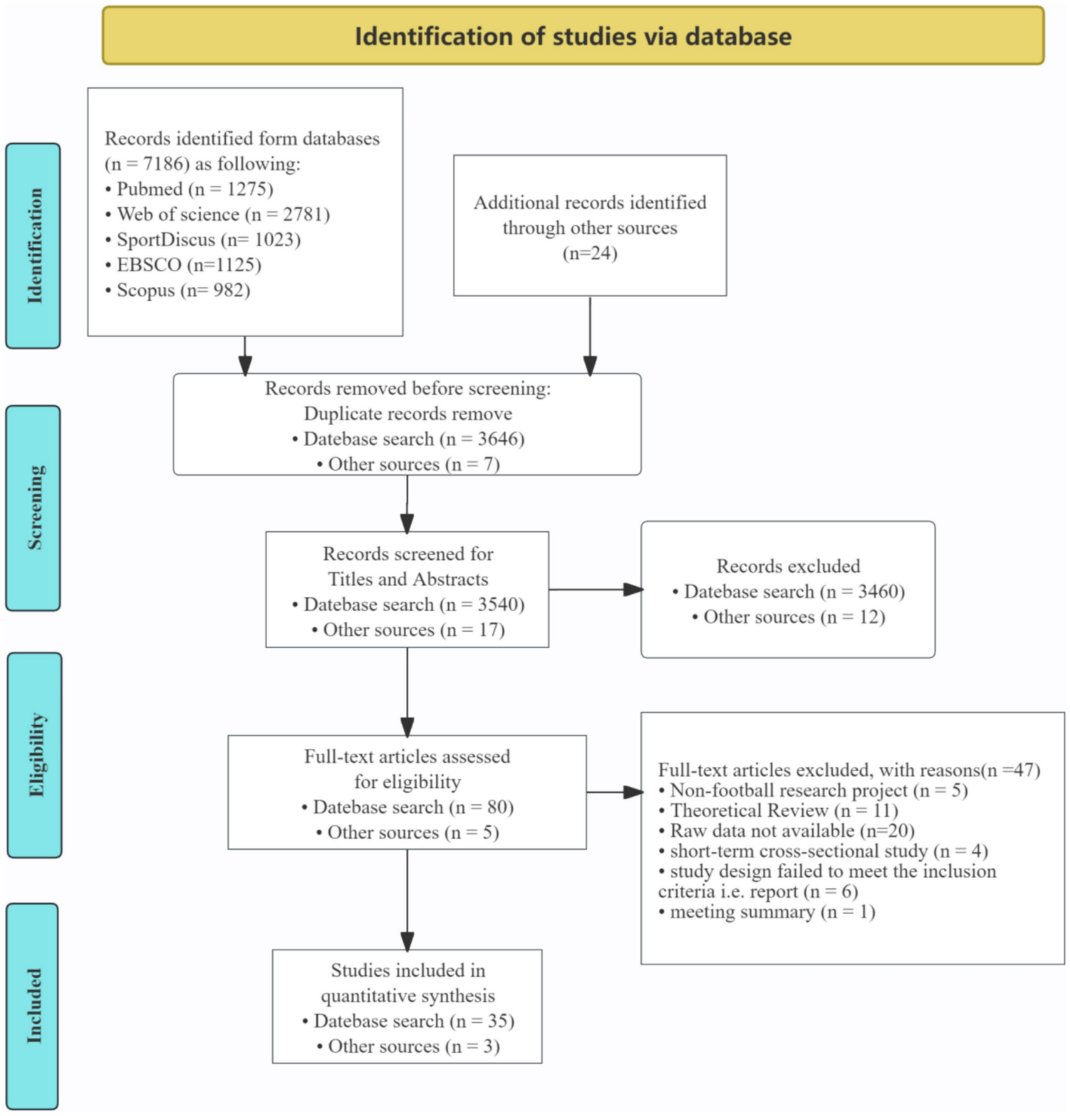
Flow diagram for screening and selection of studies according to systematic reviews (Moher et al., 2010).

Following the initial screening, the remaining 85 articles underwent a full-text review. At this stage, 47 articles were excluded due to reasons such as insufficient data, non-empirical content, methodological issues, or failure to align with the research objectives of this review. Ultimately, 38 empirical studies were retained, as they met all inclusion criteria and provided relevant, high-quality data for analysis. A detailed overview of the screening and selection process is provided in Figure 1, which illustrates the stages of record retrieval, screening, and selection.

### 3.2 Quality assessment

These studies spanned 1976–2024 and involved 15 countries or regions with a cumulative sample size of over 48,000 youth athletes (U9–U19). The quality of indicators for the included papers was as follows: (1) the mean methodological quality score for the 38 selected articles was 85%; (2) none of the articles scored below 75%; (3) all articles scored between 75 and 93.8% (see Supplementary Appendix S2). This balanced quality distribution ensured analytical reliability.

### 3.3 Basic characteristics

#### 3.3.1 Demographic features

**Figure 2:**
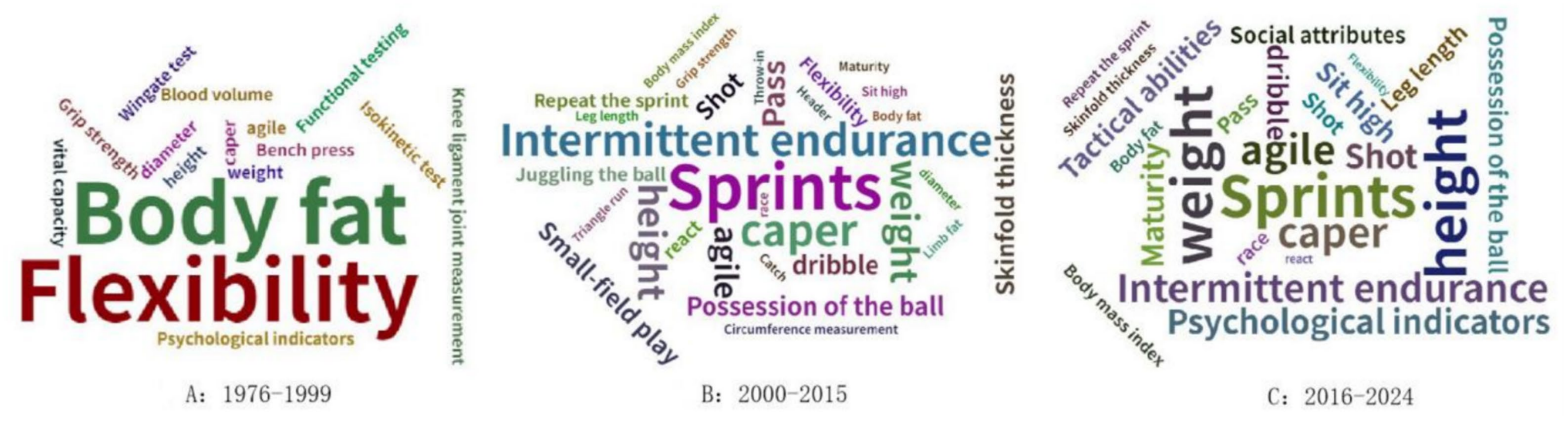
Word cloud of soccer talent identification indicators.

As Table 2, Sample sizes across the included studies varied widely, ranging from 16 to 22,843 participants. Specifically, 28.6% of studies involved fewer than 500 participants, 40.0% included samples of 500–5,000 participants, and 31.4% comprised more than 5,000 participants. The majority of studies (91.4%) focused exclusively on male adolescent populations, with only two studies conducting explicit gender comparisons. Most investigations targeted the U10–U16 age groups, with particular emphasis on U12 and U14 cohorts, underscoring their importance in early-stage talent identification. In addition, several longitudinal studies followed players for periods of up to 7 years, thereby strengthening the evaluation of long-term predictive validity (Figure 2).

**Table 2:** A summary of study characteristics of identified studies (ordered by publication date).

No	Author (s) & year	Country	Age range	Participant characteristics	Selection criterion	result
1	Raven et al. (1976)	US	19–32	*N* = 18	Anthropometry: height, weight, body fat, 7 skinfolds, and 7 diameters; Physiology: jump, flexibility, endurance run, bench press, agility run, handgrip, lung capacities, absorptive blood parameters, and aerobic endurance.	In 18 NASL pros, VO2max was 58.4 mL·kg − 1·min − 1, resting HR 50 bpm, and body fat 9.6%; 12-min run averaged 1.86 mi and Illinois agility 15.6 s. Strength/flexibility were near sedentary norms, but endurance and agility clearly distinguished players.
2	Mangine et al. (1990)	US	16–26	*N* = 83	Anthropometry: 3 skinfolds, body fat; Physiology: flexibility, knee ligament arthrometer, Wingate testing, function testing, and Isokinetic testing.	Elite US male players showed low adiposity (9.5% body fat), high anaerobic power (Wingate 8.1 W/kg), normal functional symmetry (>85%; hop 203 ± 13 cm; timed hop 1.65 ± 0.13 s), stable knees (<2.5 mm A/P in 75/76), and balanced H/Q ratios (~56% at 60°/s; ~67–70% at 450°/s); 17% had hamstring tightness.
3	Ommundsen and Vaglum (1997)	Norway	12–16	*N* = 223	Psychology: CEC, PC, PIC, and SSE.	Perceived soccer competence predicted soccer-specific self-esteem more strongly when competence was personally important; importance shifted with competence over time. Drop-out was directly linked to low perceived competence; only older players showed mediation from actual→perceived competence.
4	Junge et al. (2000)	Germany	14–41	*N* = 588	Football-Specific Characteristics; Physiology: Athletic Coping Skills Inventory, State Competitive Anxiety Test, State Trait Anger Expression Inventory; Psychology; and Reaction Time.	588 players: run shortened reaction time; high-level fastest postrun. Anxiety/anger correlated with injuries. 92% accepted fouls; unfair play tied to higher anger, poorer coping.
5	Rosch et al. (2000)	Germany	-	*N* = 588	Physiology: flexibility, sprints, three-corner run, long throw-in, long kick and aerobic endurance; Technical skills: juggle, dribble, pass, shot, and heading.	In 588 players, higher-skill groups showed better warm-up quality, speed-dribbling, power and endurance; flexibility and shooting/heading scarcely differed.
6	Hoare D and Warr (2000)	Australia	15–19	*N* = 17	Anthropometry: height and body mass; Physiology: vertical jump, sprints, 505 agility, and aerobic endurance; Technical skills: juggle, dribble, ball control, pass and receive; Match-plays: 3 v 3, 6 v 6, and full-size games.	From 59 non-soccer females (15–19y), 17 were selected via fitness/anthropometrics + coach-rated skills; after 12 months, the team went 20–3–2, with 10 reaching zone squads and 2 reaching state selection within 6 months.
7	Reilly et al. (2000)	UK	15–16	*N* = 31	Anthropometry: height, weight, 7 skinfolds, 2 diameters, and 4 girth measurements; Physiology: standing vertical jump, sprints, agility run repeated sprints, and aerobic endurance; Technical skills: shot and slalom dribble; Psychology: TEOSQ and CSAI-2.	Elite (*n* = 16) outperformed sub-elite (*n* = 15): leaner, higher VO₂max, agility/sprint, speed-endurance, vertical jump; better dribbling and 1v1 anticipation. Best discriminators: agility, 30 m sprint, ego orientation, 1v1 anticipation
8	e Silva et al. (2010)	Portugal	13.0–14.1	*N* = 128	Anthropometry: height, weight, 4 skinfolds and skeletal age; Physiology: jump, 7 sprints, RSA, agility run, and aerobic endurance; Technical skills: ball control, dribble, pass and shot; Psychology: TEOSQ.	Selected U-14 s were more biologically mature, taller/heavier, and superior in squat jump, sprint/repeated-sprint, ball control and ego orientation; no differences in agility, endurance, dribbling, shooting, passing or task orientation; position effects minimal.
9	Gonaus and Müller (2012)	Austria	14–17	N1 = 821, N2 = 3,912	Physiology: jump, sprints, hurdles agility run, aerobic endurance, foot tapping, reaction test, 2 kg medicine ball throw, and sit-and-reach.	Across U14–U17, future drafted players exceeded non-drafted in speed/power/endurance; shuttle sprint + 2-kg medicine-ball throw best predicted status (~63–66% accuracy).
10	Unnithan et al. (2012)	UK	15.4 ± 0.8	*N* = 16	Anthropometry: height and weight; Match-plays: 4 v 4; Physiology: heart rate; Technical skills: total distance covered, high speed runs, and accelerations.	Pilot 4v4 games: winners/lossers showed similar HR and running loads; game success modestly aligned with coach technical ratings (*r* = 0.39) and overall technical scores (*r* = 0.55).
11	Gil et al. (2014)	Spain	9–10	*N* = 21	Anthropometry: height, sitting height, weight, Leg length, BMI, 6 skinfolds, limb fat, and body fat; Maturity Offset; Physiology: jump, sprints, agility run, aerobic endurance, and handgrip.	U9–10: Preselected outfielders were older/leaner, faster; speed+agility best discriminated. Final picks: older, better agility+Yo-Yo IR. GKs: taller, more mature, stronger.
12	Goto et al. (2015)	UK	U9–U10	*N* = 22	Anthropometry: height and body mass; Maturity Offset; Physiology: sprints and aerobic endurance; Match-plays: 6 v 6.	U9–U10 EPL academy players ran ~4.0 km/match (~4.7 km·h − 1). Retained boys ran ~ + 0.4 km total and +0.2 km at 2.1–3.1 m·s − 1; U10 slightly higher mod/high-speed.
13	Emmonds et al. (2016)	UK	U9–U18	*N* = 443	Anthropometry: height and body mass; Physiology: sprints, and aerobic endurance.	In 443 academy males, only U16–U18 speed (10/20 m) and U18 Yo-Yo endurance distinguished later professionals; height/mass did not. Thus, use these tests for monitoring, not early talent ID.
14	Wilson et al. (2016)	UK	13.6–18.5	*N* = 40	Technical skills: juggle, pass, shot, wall-pass, dribble and rebound.	Eight skill tests were highly repeatable (ICC 0.83–0.95); composite skill ICC 0.98. PCA1 (46.9%) indexed overall skill; skills covaried positively and rose with age.
15	Höner and Votteler (2016)	Germany	11.4 ± 0.3	*N* = 22,843	Anthropometry: height and weight; Physiology: sprints and agility run; Technical skills: dribble, shot and ball control; Score: negatively coded tests.	In 22,843 U12 elites, motor score predicted U16–U19 selection (all tests *p* < 0.01) but individual validity was low; PR ≥ 99 raised youth NT odds ×12; higher cutoffs ↑specificity, ↓sensitivity.
16	Höner and Feichtinger (2016)	Germany	U12	*N* = 2,677	Physiology: sprints and agility run; Technical skills: dribble, shot, and ball control; Psychology: AMS-S, SOQ, TEOSQ, VCS, PSC, SES and CAI-T.	Among 2,677 U12 elites, only self-referential cognitions related meaningfully to motor score (r = 0.10–0.37). Ten traits predicted U16 academy selection (OR = 1.61–2.65).
17	Fenner et al. (2016)	UK	U10	*N* = 16	Anthropometry: height and weight; Physiology: jump and sprints; Match-plays: 4 v 4; Score: GTSC and TP.	U10 elites (*n* = 16): SSG points closely matched coach technical scores (*r* = 0.758); higher scores also related to greater total and high-speed distance, not sprint/jump tests.
18	Hirose and Seki (2016)	Japan	13.0–13.9 15.0–15.9	N1 = 38 N2 = 20	Anthropometry: height, sitting height, and weight; Maturity Offset; Physiology: 5-step bounding, sprint, and 10 m × 5 COD.	Over 2 years, height/weight and 40-m sprint rankings were stable (*r* = 0.80–0.94), but COD rankings were not; thus sprint speed may aid talent ID, whereas power/COD are too changeable.
19	Woods et al. (2016)	Australia	U18	*N* = 55	Match-plays: full-size games, total distance, relative distance, high speed running distance, total disposals, marks, contested possessions, uncontested possessions, inside 50s and rebound 50s; Score: GEE.	Drafted U18 AF players had markedly more contested possessions and inside-50 entries; GEE identified both as key predictors of draft outcome, while GPS running metrics were non-discriminative.
20	Leyhr et al. (2018)	Germany	U12–U15	*N* = 1,134	Physiology: sprints (20-m) and agility run; Technical skills: dribble, shot, and ball control; Score: negatively coded tests; Criterion: APL.	*n* = 1,134 (U12–U15): motor tests improved nonlinearly. Future elites were better in motor score, agility and skills (dribbling/ball control/shooting), not sprint; slopes did not predict adult level.
21	Höner et al. (2017)	Germany	U12	*N* = 14,178	Anthropometry: height, sitting height, and weight; Physiology: sprints (20-m) and agility run; Technical skills: dribble, shot, and ball control; Criterion: APL.	In 14,178 U12s, all five motor tests and size (not relative age) predicted adult level; SEM explained 25% variance, with technical skills > speed for later success.
22	Tribolet et al. (2018)	Australia	U13–U15	*N* = 277	Anthropometry: stature, sitting height, body mass, leg length, and maturity offset (APHV); Physiology: KTK-3, change of direction speed (T-test), and standing broad jump; Technical skills: kicking and handballing.	277 U13–U15 AF: age increased size, COD speed, power and coach-rated skills; motor competence unchanged. U15 selections favored earlier maturing, larger, faster COD, more powerful, better kick/mark; 90% classified.
23	Peña-González et al. (2018)	Spain	U12–U16	*N* = 564	Anthropometry: height, sitting height, leg length, and body mass; Maturity Offset (APHV); Physiology: jump (CMJ), sprints (30-m), agility run, and aerobic endurance (Yo-Yo IR1); Score: CEE.	564 U12–U16 boys: Q1 births overrepresented. Adjusted for CA and maturity, size/fitness did not vary by quartile, but coaches expected more from Q1 than Q4.
24	Bennett et al. (2019)	Australia	8.0–16.9	*N* = 328	Match-plays: attacking situations (2 v 1, 3 v 1, 3 v 2, 4 v 3, and 5 v 3); Score: video-based decision-making assessment.	In 328 youths, an iPad video test showed partial construct validity (older = faster; complexity ↓accuracy/↑time) but weak academy discrimination; only accuracy modestly separated controls.
25	Bidaurrazaga-Letona et al. (2019)	Spain	12.3 ± 0.3 14.0 ± 0.2	N1 = 50 N2 = 44	Anthropometry: height, sitting height, leg length, body mass, BMI, 6 skinfolds (triceps, subscapular, abdominal, suprailiac, thigh, and calf), body fat, and maturity offset (APHV); Physiology: jump (CMJ), sprints (15-m), agility run, and aerobic endurance (Yo-Yo IR1).	U13 retention linked to superior & improving fitness (speed/agility/endurance). U15 entry favored older, larger, more mature players; club players’ gains narrowed gaps. Strong RAE (few Q4 births).
26	Platvoet et al. (2020)	Netherland	U11	*N* = 103	Anthropometry: height and weight; Physiology: KTK, sprints (15-m/30-m) and agility run; Technical skills: dribble and LSPT; Psychology: SISP.	Selected U11s outperformed deselected peers in slalom sprint, dribbling and passing, plus higher coach-rated sport-learning, creativity, motor & interpersonal capacity; no differences in age, anthropometrics or KTK coordination; DA classified ~66%.
27	Dugdale et al. (2021)	UK	8.0–17.0	*N* = 537	Anthropometry: stature and body mass; Physiology: jump (CMJ), sprints (5-m/10-m/20-m), and aerobic endurance (Yo-Yo IR1).	Only 10% earned contracts; early recruitment did not increase success. Successful players lagged physically until ~13–14y but improved faster, later outperforming in sprint/YYIRT/CMJ; stature/mass were weak indicators.
28	Abidin (2021)	Turkey	U13		Technical skills: 18 criteria; Score: scoring system of Hit/it and coaches observe; Classification: ML algorithms.	In 537 academy players, 10% gained a club pro contract; recruitment age did not raise odds. Physical/fitness superiority emerged only from ~13–14y onward.
29	Wilson et al. (2021)	Brazil	10.9–12.8, 10.6–13.2, 14.3–15.1	N1 = 42, N2 = 42, N3 = 26	Match-plays: 3 vs. 3; Technical skills: total number of individual goals, total number of goals scored by their team-mates, total number of goals scored by their team, total number of goals conceded by their team, and total net goals for the team. Score: PAC.	In randomized 3v3 SSGs, net-goal performance was repeatable (ICC = 0.57–0.69). PCA separated overall success (PC1) and contribution type (PC2). Simulations: ~28 games for R^2^ ≥ 0.80.
30	Höner et al. (2021)	Germany	U12–U15	*N* = 13,869	Physiology: sprints (20-m) and agility run; Technical skills: dribble, ball juggling, and ball control; Tactical skills: behavior in offensive situations, behavior in defensive situations, game intelligence; Psychology: motivational skills, volitional skills and social skills.	Future academy-selected players outperformed others on all 9 predictors (*p* < 0.001); combined subjective+objective model best predicted success (Nagelkerke R^2^ = 0.15–0.20), with sprint, tactical skills, dribbling strongest.
31	Konarski et al. (2021)	Poland	13.6–15.3	*N* = 31	Anthropometry: height, sitting height, leg length, FFM, muscle mass, body fat, 4 skinfolds, and maturity offset; Physiology: vertical jump, sprints, agility run, aerobic endurance, and handgrip. Technical skills; Tactical skills: behavior in offensive situations, behavior in defensive situations, creativity and decision making, and overall effectiveness in play.	Select U15s were more biologically mature and showed higher jump/grip strength and coach-rated attacking tactics/creativity; these best discriminated selection (84–86%).
32	Irurtia et al. (2022)	China	8.0–13.9	*N* = 722	Anthropometry: height, body mass, and BMI; Physiology: jump (SJ, CMJ, and ABK), sprints (10-m /30-m), RSS, SRT, and FVC.	Talented U8–U13 Chinese players showed better jump/sprint/agility, but fitness/anthropometry poorly predicted match ranking (MLR R^2^ = 0.05–0.20); motor tests should not drive talent ID alone.
33	Lethole et al. (2024)	South Africa	13–18	*N* = 173	Physiology; Technical skills; Tactical skills; Perceptual-cognitive; Psychology, and Social attributes.	Coaches’ ratings clustered into 6 dimensions (PCA: 68.96% variance): psychological, physical, social, technical, tactical, and perceptual-cognitive; top attributes were concentration/bravery, coachability, and decision-making.
34	Altmann et al. (2024)	Germany	U12–U19	*N* = 409	Physiology: jump, sprints (30-m), aerobic capacity and aerobic speed reserve. Technical skills: total score; Psychology: MPS, MPS pressure/stress, MPS structure, MPS attention, MPS competition, SPS, SPS positive minus negative, determination test of the vienna test system, and cognitive flexibility.	XGBoost predicted next-year selection with ROC-AUC = 0.69 (F1 = 0.84); 30 m sprint, Footbonaut skill, CMJ and aerobic speed reserve dominated; psychology was moderate.
35	Toselli et al. (2024)	Italy	U10–U12	*N* = 78	Anthropometry: height, sitting height, leg length, body mass, limb perimeters, body fat, BMI, and maturity offset; Physiology: jump (CMJ), sprints (15-m), RSA, and Harre test.	Selected U10–U11 players were leaner/faster; overall RAE/MS not different. LDA: RSA, earlier APHV, and larger humeral width best discriminated selection (Wilks’ *λ* = 0.76; 42% selected, 85% unselected).
36	Dugdale et al. (2021)	UK	16–18	*N* = 20	Match-plays: 4 vs. 4; Psychology: coachability, adaptability, decision making, positive attitude, resilience, X-factor, competitiveness, confidence, maintaining composure under pressure, match presence, communication, on pitch bravery and anticipation.	HSBST showed strong content/face validity (practitioners prioritized resilience/competitiveness/decision-making) and acceptable reliability using dichotomous scoring: inter-rater 81.3–89.9%, intra-rater 80.4–99.4%.
37	Hirose and Seki (2016)	Japan	U13\U15	*N* = 37	Anthropometry: height, sitting height, and weight; Maturity Offset (APHV); Physiology: 5-step bounding, sprint (40-m), and 10 m × 5 COD test.	Two-year rank tracking was strong for 40-m sprint (*r* = 0.81–0.90) and size (*r* = 0.80–0.94), moderate for 5-step bounding (*r* = 0.48–0.64), but absent for 10 × 5 COD; sprint speed is best talent-ID index.
38	Höner and Votteler (2016)	Germany	U12	*N* = 22,843	Anthropometry: height and weight; Physiology: sprint (20-m), and agility run; Technical skills: dribble, shot, and ball control; Predictors in U16–U19.	Motor score predicted U16–U19 selection (all tests *p* < 0.01) but individual validity was low; PR ≥ 99 raised youth NT odds ×12; higher cutoffs ↑specificity, ↓sensitivity.

VO2max, maximal oxygen uptake; VE, pulmonary ventilation; HR = heart rate; CEC, coach-evaluated soccer competence; PC, perceived soccer competence; PIC, perceived importance of soccer competence; SSE, soccer-related self-esteem; TEOSQ, Task and Ego Orientation in Sport Questionnaire; CSAI-2, Competitive State Anxiety Inventory-2; SA, estimate skeletal age; Yo-Yo IR1, Yo-Yo intermittent endurance test level 1; BMI, body mass index; APHV, age at peak height velocity; CMJ, counter movement jump; PHV, peak height velocity; Yo-Yo IE2, Yo-Yo intermittent endurance test level 2; AMS-S, Achievement Motives Scale-Sport; SOQ, Sport Orientation Questionnaire; VCS, Volitional Components in Sport; PSC, Physical Self-Concept Scales; SES, Self-Efficacy in Soccer; CAI-T, Competition Anxiety Inventory Trait; GTSC, game technical scoring chart; TP, total points; COD, change of direction; GEE, generalized estimating equations; APL, adult performance level; KTK, Körperkoordinations Test für Kinder; CEE, coaches’ efficacy expectations; LSPT, Loughborough Soccer Passing Test; SISP, Scale for Identification of Sport Potential; ML, machine learning; PAC, principal component analysis; FFM, fat-free mass; SJ, squat jump; ABK, Abalakov; RSS, repeated side-step; SRT, sit & reach test; FVC, forced vital capacity; SPS, stress profile sport; MPS, motive profile sport; RAE, relative age effect; and RSA, repeated sprint ability.

#### 3.3.2 Temporal distribution

Research on soccer talent identification (TID) has progressed through three distinct phases: (1) Early Stage (1976–1999): During this period, talent identification was largely based on subjective judgment, intuition, and observational methods. The few studies available primarily focused on physical attributes, such as speed, strength, and stamina, which were considered primary indicators of soccer potential. The emphasis was on assessing basic physical fitness and technical skills in young athletes. (2) Development Stage (2000–2010): Research in this phase became more methodologically rigorous, incorporating structured testing to evaluate players’ performance in areas such as agility, ball control, passing accuracy, and decision-making under pressure. Standardized fitness assessments, including the Yo-Yo intermittent recovery test and the beep test, were widely used to assess cardiovascular endurance. Furthermore, studies began integrating more advanced physiological assessments, such as heart rate variability and lactate threshold, to identify athletes’ capacity to maintain high-level performance in competitive environments. (3) Active Stage (2011–2024): Characterized by a significant increase in publications, this phase saw an expanding focus on multidimensional modeling and the integration of advanced technologies. Innovations such as artificial intelligence (AI), GPS tracking systems, and biological maturity estimation became widely adopted, greatly enhancing the precision and diversity of talent evaluation.

#### 3.3.3 Geographic distribution

The 38 included studies were drawn from 15 countries or regions, with a geographically uneven distribution. Europe contributed 54.3% of the evidence base (predominantly Germany, the United Kingdom, and Spain), followed by Asia at 25.7% (primarily China and Japan) and Oceania at 11.4% (mainly Australia); the Americas and Africa accounted for 5.7 and 2.9%, respectively. This distribution aligns with marked regional heterogeneity in TID logics, indicator composition, and technical pathways.

Clear region-specific emphases were observed. European studies largely prioritized the implementation of standardized metric frameworks and longitudinal tracking designs, reflecting a strong orientation toward structured, scalable assessment systems. In contrast, Asian studies more frequently emphasized localized model development, integrating technical competencies with physical conditioning and culturally adapted testing to support early-stage talent identification (e.g., localized integration approaches and test adaptation). Taken together, these findings underscore substantial diversity in regional talent selection paradigms, suggesting that no single TID model is likely to be universally optimal across contexts.

#### 3.3.4 Methodological features

All included studies reported specific selection indicators and corresponding testing methods. The majority of studies covered a research period exceeding 5 years, with eight identified as longitudinal investigations spanning 7 years or more. The assessed dimensions were classified into six categories: physical characteristics, physiological development, physical fitness, technical performance, psychological traits, and social adaptation, comprising more than 60 distinct indicators. Most studies adopted multidimensional assessment frameworks, typically integrating at least three domains (e.g., “physical + fitness + technical”), reflecting an increasing trend toward indicator integration in soccer talent identification. Furthermore, several high-quality studies employed advanced analytical techniques, such as principal component analysis, machine learning algorithms, and predictive modeling, thereby accelerating the transition of talent identification toward a data-driven paradigm (Abidin, 2021).

## 4 Discussion

This systematic review synthesizes evidence on the evolution of soccer talent identification and development (TID) metrics and identifies three overarching patterns. First, technological advancement has broadened the scope and objectivity of assessment by enabling multi-indicator profiling and increasingly standardized workflows. However, this expansion has also tended to privilege variables that are readily quantifiable, potentially constraining the construct space toward measurement convenience rather than match-determining performance qualities. Second, although intensified global competition has promoted standardization, this process has not resulted in global homogenization. Instead, TID logics remain distinctly region-specific, shaped by differences in football culture, developmental infrastructures, and access to resources and technology. Third, the field continues to be shaped by unresolved tensions related to fairness—most notably maturity-related bias—the limited operationalization of psychosocial constructs, and unequal technological access. Collectively, these factors restrict the validity, equity, and cross-context transferability of contemporary TID systems.

Accordingly, this Discussion is organized into three sections. Section 4.1 interprets the core drivers underpinning the evolution of TID metrics, with a focus on technological advancement, competitive pressures, and regional contexts. Section 4.2 critically examines persistent challenges and contradictions that undermine validity and fairness in current practice. Section 4.3 translates the synthesized evidence into research- and practice-oriented guidelines, emphasizing human–machine collaboration, biological maturity correction, cross-regional integration, ethical governance, and longitudinal dynamic assessment.

### 4.1 Core factors driving the evolution of soccer TID metrics

Technological advancement represents a central driver of innovation in TID metrics, primarily by enhancing measurement precision, improving reliability, and enabling the integration of heterogeneous indicators into decision-making processes. The widespread adoption of sensor-based systems, player-tracking technologies, and digital platforms has facilitated more objective physical profiling and increasingly granular monitoring of performance-related outputs. In parallel, advances in machine learning and AI-based analytics have made it more feasible to combine multiple dimensions—such as physical, technical, and tactical proxies—and align them with positional or role-specific demands, potentially improving operational efficiency within selection and development pathways.

However, technology does not merely improve measurement accuracy; it also implicitly shapes how “talent” is operationalized within identification systems. When the availability of a metric becomes the primary rationale for its inclusion, identification frameworks risk drifting toward what is easiest to measure rather than what is most developmentally or competitively decisive. As a consequence, context-dependent competencies—such as perceptual–cognitive skills, tactical awareness, and creative decision-making—may remain underrepresented, despite their centrality to match performance. Technological progress should therefore be viewed as both an enabler and a potential source of systematic bias: while it enhances reliability for certain outputs, it may simultaneously amplify construct underrepresentation for complex, emergent behaviors that are difficult to quantify.

Global competition has further accelerated calls for standardization and comparability across academies and regions. Nevertheless, the evidence synthesized in this review indicates that standardization does not equate to homogenization. Regional cultural and developmental contexts continue to shape distinctive TID logics. European systems often emphasize data-driven precision and structured evaluation frameworks; South American and African contexts tend to prioritize the identification of latent talent expressed in game-like environments, such as creativity and decision-making under pressure; and Asian systems frequently focus on localized integration and culturally adapted testing pathways. These patterns suggest that TID metrics are best conceptualized as context-calibrated instruments rather than universally invariant standards. The same test or indicator may not carry equivalent construct meaning, developmental relevance, or predictive validity across different environments.

Section takeaway. The evolution of TID metrics reflects a co-development of technological capability and standardization pressure, with regional context playing a decisive moderating role in what is measured and how results are interpreted. A key methodological gap is that cross-context comparability is often assumed rather than empirically tested through validation or calibration studies, thereby limiting the robustness of global benchmarking efforts.

### 4.2 Current challenges and contradictions in TID

Despite advances that have increased the apparent scientific rigor of TID systems, several persistent contradictions continue to constrain real-world validity and fairness.

First, the “double-edged sword” of datafication has become increasingly evident. Over-reliance on quantifiable indicators can lead to selection systems that disproportionately reward measurable outputs while overlooking players who influence game dynamics through less easily captured mechanisms, such as off-ball intelligence, anticipation, or creative problem-solving under pressure. Although objective technologies improve measurement precision, they do not inherently guarantee construct validity. The principal risk is therefore not measurement error per se, but construct omission—failing to measure attributes that are critical to long-term performance development.

Second, fairness issues related to biological maturity remain a structural source of bias, particularly during early adolescence. TID systems that rely predominantly on chronological age grouping tend to advantage early-maturing players through transient physical benefits (Dodd and Newans, 2018), while systematically undervaluing late-maturing players who may possess substantial long-term technical or tactical potential. In this sense, many selection decisions inadvertently conflate current developmental status with stable talent potential (Towlson et al., 2022). Without explicit correction mechanisms, such practices risk premature deselection and avoidable talent loss, especially within the U12–U14 age range.

Third, the operationalization of psychological and social indicators remains methodologically challenging. Although questionnaires and coach ratings enhance feasibility, they are susceptible to subjective bias, limited sensitivity to complex behaviors, and uncertain cross-cultural transportability. Consequently, psychosocial constructs are frequently incorporated into multidimensional frameworks, yet the evidentiary basis supporting their measurement quality—and their incremental predictive value beyond physical and technical indicators—remains insufficient in many cases.

Finally, unequal access to advanced technology exacerbates global disparities in TID practice. High-resource environments are able to deploy sophisticated tools and data infrastructures, potentially improving identification efficiency and developmental monitoring, whereas low-resource contexts often rely on traditional methods. This technological divide is not merely logistical; it shapes the dominant evidence base and can reinforce a hierarchy of “best practice” that is difficult to adopt universally.

Section takeaway. Contemporary limitations in TID increasingly reflect governance and interpretation challenges—what is measured, how indicators are weighted, and whether systems operate fairly across developmental and cultural contexts—rather than a simple lack of available tests. Addressing these contradictions requires methodological rigor, including validation, calibration, and longitudinal designs, alongside explicit ethical oversight.

### 4.3 Guidelines for research and practice

Human–machine collaboration within a dynamic assessment framework.

Optimizing TID requires a dynamic framework that balances objective data with structured expert judgment. As indicator systems become more complex and analytical tools more sophisticated, the central challenge shifts from data accumulation to meaningful integration without sacrificing ecological validity. Excessive reliance on automated outputs may obscure unstructured or emergent potential, whereas purely experience-based decisions can lack consistency and transparency. A defensible direction is a human–machine collaboration model in which coaches’ expertise is formalized through structured observation protocols and systematically cross-validated against objective indicators and algorithmic outputs. Practically, this may include standardized observation templates for tactical and creative behaviors, inter-rater reliability monitoring, and routine audits of cases in which expert judgment diverges from model predictions.

Embedding biological maturity correction as a core fairness mechanism.

Integrating biological maturity correction into routine assessment is essential for safeguarding fairness and enhancing long-term predictive validity. Maturity-related variability should be treated as a structural confound rather than random noise, particularly during early adolescence. Correction procedures should be embedded within the core workflow of TID systems, coupled with longitudinal monitoring to accommodate non-linear developmental trajectories and to avoid overconfidence in single time-point rankings.

Cross-regional integration toward a globally compatible model.

Promoting cross-regional integration represents a strategic pathway toward a globally compatible, rather than globally uniform, TID framework. Evidence suggests that different regions emphasize complementary strengths in identification logic and system design. A future-compatible model should integrate these strengths through mechanism-level adaptation rather than surface-level replication. In practice, effective transfer requires an adapt–pilot–validate cycle, ensuring that constructs, thresholds, and interpretations are calibrated to local developmental ecologies before broader implementation.

Technology accessibility and ethical governance.

Enhancing technology accessibility and ethical governance is critical for the sustainable evolution of TID. Priorities include the development of low-cost, scalable toolchains to reduce entry barriers, and the establishment of ethical frameworks addressing data privacy, scoring fairness, and safeguards against implicit discrimination. As AI-enabled models become more prominent, transparency and interpretability are prerequisites for accountability and trust.

Long-term tracking and dynamic re-evaluation.

Finally, long-term tracking and dynamic re-evaluation should be institutionalized as core principles of TID. Talent development is best conceptualized as a dynamic process requiring repeated assessment and periodic recalibration, rather than as the outcome of static, one-off evaluations. Longitudinal monitoring allows for more accurate interpretation of growth-related variability and reduces the risk of premature selection or deselection decisions.

Overall Conclusion.

Collectively, the evidence supports a conceptual shift in soccer TID from static, tool-driven testing toward governance-oriented, context-calibrated systems. Such systems integrate objective technologies with structured expert judgment, correct for maturity-related bias, facilitate cross-regional learning through validated adaptation, ensure ethical and accessible technology deployment, and embed longitudinal dynamic assessment as the foundation for sustainable decision-making.

### 4.4 Limitations

Several limitations should be acknowledged. First, this review included only English-language publications, which may have led to the underrepresentation of region-specific evidence from non–English-speaking countries. This language restriction is particularly relevant when interpreting findings framed as “global,” as it may bias conclusions toward English-dominant and high-resource contexts. Second, heterogeneity in study designs, indicators, and developmental stages limited direct cross-regional and longitudinal comparability; thus, the proposed framework reflects dominant trends rather than causal relationships. Third, this review was not registered in PROSPERO, which may reduce transparency regarding protocol deviations. Future reviews would benefit from preregistration, multilingual search strategies, and broader regional coverage to strengthen global generalizability.

## 5 Conclusion

This review indicates that the evolution of soccer talent identification and development (TID) metrics has been primarily driven by technological progress and increasing global competition. Tools such as sensors, GPS-based tracking, and multidimensional evaluation frameworks have expanded the range of quantifiable indicators and facilitated more standardized assessment workflows. Europe has played a leading role in advancing such standardization (e.g., longitudinally informed integrated scoring approaches), which has also influenced talent selection practices in other regions. Nevertheless, standardization has not resulted in homogenization. Distinct regional pathways persist in both selection logic and metric emphasis: European systems tend to prioritize data-driven precision and procedural standardization, whereas South American and African contexts often place greater value on identifying latent talent and capturing context-dependent performance in game-like situations.

At the same time, the available evidence highlights a “double-edged” effect of datafication in TID. Over-reliance on measurable outputs may mask creativity and other decisive behaviors under high-pressure conditions, while GPS and similar tools have inherent limitations in representing intuitive decision-making and tactical awareness. Furthermore, maturity-related bias remains a structural determinant of fairness and long-term predictive validity, particularly in the U12–U14 age range. Early-maturing players may be systematically overvalued due to temporary physical advantages, whereas late-maturing players may be underestimated or prematurely deselected despite developmental potential. Incorporating maturity-adjustment approaches (e.g., PHV/APHV-based correction) is therefore a necessary step toward improving both equity and predictive accuracy in youth TID systems.

Although theoretical frameworks such as the DMGT (Gagné, 2004) and DMSP (Cote et al., 2004) provide valuable insights into talent development processes, the multidimensional and context-dependent nature of football performance, together with pronounced geo-cultural heterogeneity across Europe, South America, and Asia, complicates the integration of a single, universally applicable TID framework. Findings from this synthesis further suggest that standardization within TID does not imply homogenization; rather, the operationalization of selection criteria is strongly shaped by region-specific training ecosystems and culturally embedded value systems. Consequently, future research and practice should move beyond one-size-fits-all models and advance an “inclusive and adaptive” framework that achieves global coherence while preserving the diversity of regional football cultures.

## Data Availability

The original contributions presented in the study are included in the article/Supplementary material; further inquiries can be directed to the corresponding author/s.
