# Supplementary material for: Evolution of soccer talent identification criteria: a systematic review from global perspectives (1976–2024)
Source: Front Psychol. 2026 Mar 19;17:1739010. doi: 10.3389/fpsyg.2026.1739010 (PMC13045062; doi:10.3389/fpsyg.2026.1739010)
Supplement: Supplementary file 1 [file Table_1.DOCX]

Electronic Supplementary Material Table S1 - Quality criteria used to analyse the quantitative publications (Sarmento H. et al.,2018)

| *Q1* | Was the study purpose stated clearly? | 1=Yes | 0=No |
| --- | --- | --- | --- |
| *Q2* | Was relevant background literature reviewed? | 1=Yes | 0=No |
| *Q3* | Was the design appropriate for the research question? | 1=Yes | 0=No |
| *Q4* | Was the sample described in detail? | 1=Yes | 0=No |
| *Q5* | Was sample size justified? | 1=Yes | 0=No |
| *Q6* | Was informed consent obtained? (if not described, assume No) | 1=Yes | 0=No If not applicable, assume NA |
| *Q7* | Were the outcome measures reliable? (if not described, assume No) | 1=Yes | 0=No |
| *Q8* | Were the outcome measures valid? (if not described, assume No) | 1=Yes | 0=No |
| *Q9* | Was method described in detail? | 1=Yes | 0=No |
| *Q10* | Were results reported in terms of statistical significance? | 1=Yes | 0=No |
| *Q11* | Were the analysis methods appropriate? | 1=Yes | 0=No |
| *Q12* | Was importance for the practice reported? | 1=Yes | 0=No |
| *Q13* | Were any drop-outs reported? | 1=Yes | 0=No If not applicable, assume NA |
| *Q14* | Were conclusions appropriate given the study methods? | 1=Yes | 0=No |
| *Q15* | Are there any implications for practice given the results of the study? | 1=Yes | 0=No |
| Q16 | Were limitations of the study acknowledged and described by the authors? | 1=Yes | 0=No |

NA- Not applicable
